# Self-medication among pregnant women in comparison to the general population: a scoping review of the main characteristics

**DOI:** 10.61622/rbgo/2024rbgo77

**Published:** 2024-12-04

**Authors:** Gabriela Pereira, Cinthia Madeira de Souza, Amanda Canato Ferracini, Fernanda Garanhani Surita, Sherif Eltonsy, Priscila Gava Mazzola

**Affiliations:** 1 Universidade Estadual de Campinas Campinas SP Brazil Universidade Estadual de Campinas, Campinas, SP, Brazil.; 2 University of Manitoba Rady Faculty of Health Sciences College of Pharmacy Canada College of Pharmacy, Rady Faculty of Health Sciences, University of Manitoba, Canada.

**Keywords:** Self-medication, Medication use, Pregnant women, Drug-related side effects and adverse reactions

## Abstract

**Objective:**

An in-depth evaluation of the published evidence is needed on self-medication, specifically the evidence focusing on vulnerable groups, such as pregnant women. This scoping review aims to provide an overview of the differences in self-medication prevalence and study characteristics among different groups, while identifying gaps in the literature.

**Methods:**

A literature search was performed in PubMed and Web of Science, including articles published in the last 10 years for the pregnant women group (PWG) and the general population group (GPG). Data on study design, self-medication prevalence, medications used, and other variables were collected, tabulated, and summarized.

**Results:**

From 2888 screened articles, 75 were considered including 108,559 individuals. The self-medication (SM) in the PWG ranged from 2.6 to 72.4% and most studies had an SM prevalence between 21 and 50% and in the GPG, 32 from 50 studies had a SM prevalence higher than 50%. The reviewed studies varied considerably in methodology, requiring careful interpretation. While most of the studies assessed self-medication during the entire pregnancy, self-medication definition was often inconsistent between studies. Acetaminophen was the most used medication and headache was the most frequent symptom leading to self-medication initiation in the PWG.

**Conclusions:**

Self-medication among pregnant women showed a lower prevalence when compared to the general population. The medications used and symptoms reported were similar between groups. However, methodological differences must be carefully considered. Pregnant women should carefully follow their physicians’ advice before initiating self-medication to avoid preventable maternal and fetal adverse effects.

## Introduction

Self-medication (SM) was defined by the World Health Organization (WHO) in 1998 as the "selection and use of medicines by individuals to treat self-recognized illness or symptoms",^(1)^ including the use of herbal and medicinal products. However, the definition of SM can be broader, and some authors consider the use of previous prescriptions, the administration of a medicine prescribed to a family member or just the administration of any over the counter (OTC) drug as SM.^(2,3)^

SM practice is considered part of self-care when it is practiced in a responsible way.^(1)^ However, in many instances in developing countries, SM plays a key role as the main approach patients use in the treating their health problems.^(4,5)^ This practice is driven by different factors, which may prevail according to the sitting.^(6–10)^

Due to specific physiological characteristics, children, elderly and pregnant women are vulnerable populations with special medication use restrictions. Generally, those population groups are not often included in clinical trials, thus increasing the possibility of being exposed to unknown risks.^(11)^ Even when the safety profile is assessed in pre-clinical studies, it is important to be aware of the possible risks. One example is thalidomide tragedy, which despite the safety profile presented in pre-clinical trials, caused several birth defects among thousands of infants from mothers who used this medication during the susceptible pregnancy period.^(12,13)^

SM among pregnant women can be associated with an increased risk of drug related problems (DRP), adverse drug reactions (ADR), incorrect diagnoses and other problems related to drug therapy.^(14–16)^ The prevalence of SM among pregnant women depends on different factors, including the cultural behaviors to medications use across different countries. However, less is known about this behavior before pregnancy and the differences in comparison to the general population.

Considering the volume of publications in this field and the need to understand SM behavior among different populations, we aimed to provide an overview of the main points related to self-medication practice and studies characteristics, as well as to identify gaps in the literature that could be addressed to improve self-prescribing among pregnant women and general population.

## Methods

This review follows the recommendations of Arksey and O’Malley and the PRISMA extension for scoping reviews.^(17,18)^ The review protocol was registered at "OSF Registries" and can be accessed through: (Registration DOI 10.17605/OSF.IO/JERY7 https://doi.org/10.17605/OSF.IO/JERY7). Patient, Intervention, Comparison and Outcome (PICO) strategy was used; namely: "population" as pregnant, puerperal and nonpregnant women that became pregnant in the last few years, "intervention" as self-medication during pregnancy, "comparison" as self-medication in general population and "outcome" as the prevalence of self-medication, medications used, symptoms reported and the recording period.

The literature search was conducted from September 2019 to March 2020 in the following databases: Pubmed, PubMed Central and Web of Science. Two search strategies were performed according to each group (pregnant women and general population). The Medical Subject Headings (MeSH) terms selected to Pregnant Women Group (PWG) were *self-medication, pregnancy* and *pregnant women*; and the search strategy used was "Self Medication" AND (Pregnancy OR "Pregnant Women"). The search used included articles published in the last 10 years

The MeSH terms selected to General Population Group (GPG) were: *adolescents, young adult, adult, middle aged, aged* and *aged, 80 and over*; and the search strategy used was "Self Medication" NOT ("Self Medication" AND (Pregnancy OR "Pregnant Women"). The Boolean operator ‘’NOT" was used to separate the groups and exclude pregnant women from general population strategy. A librarian was consulted to develop the search strategy and ensure completeness of retrieved data.

We included original articles published in the last 10 years in English, Spanish and Portuguese. Systematic reviews and meta-analyses, qualitative studies, case reports and conference abstracts were excluded. Mixed methods studies (for instance a cross-sectional study with qualitative section study) were included when the quantitative section fitted our inclusion criteria. Studies performed with a population of health professionals and/or health science students (e.g. pharmacists, nurses, physicians) were not included since their background in medical sciences could have influenced the results.

For definition purposes, this review considered studies performed to evaluate the use of OTC and the practice of self-medication performed with prescription only medicines without a medical indication.

Several studies on self-medication during pregnancy considered pregnant women under 18 years old, therefore no minimum age limit was included in this review. While for the general population group, we considered only studies with participants older than 18 years. For the purpose of the scoping nature of this review, the MeSH term "adolescents" was used in our search strategy, which could include people 18 years of age. For feasibility reasons, we did not include articles discussing self-medication among specific populations with special medical conditions/diseases.

The information was recorded in a database regarding the following topics: author(s), year and country of publication, study design, recording period considered to assess the prevalence of self-medication, self-medication prevalence, medications used, symptoms reported, study aims and conclusion. The results were separated according to the continent of publication and the income was assed considering the data available in "The World Bank" list. To assess SM prevalence, we reported the definition of SM used in each study. The results are summarized in chart 1 (PWG) according to continent of publication.^(2,19–39)^

**Chart 1:** Results from self-medication (SM) practice in the Pregnant Women Group (PWG) according to continent of publication.

Author, Year, Country	Sample size	Study aims	Exposure period	SM prevalence n(%)	Medications used n(%)	Related symptoms n(%)	Comments and general Conclusion
**CONTINENT - AFRICA**							
Abasiubong et al. (2012)^(19)^ Nigeria	518 pregnant women, from 18 to >40 years	Evaluate the extent, nature and factors involved in SM among PW	Anytime during pregnancy	375(72.4)	Analgesics 157(30.3), antibiotics 138(26), herbs and other drugs 47(9.1) and sedatives 15(2.9)	-	SM is common among PW and there is need of education about potential harms to mother and fetus
Abeje et al. (2015)^(20)^ Ethiopia	510 pregnant women from 15 to 42 years	Assess SM practice and factors associated among PW	Anytime during pregnancy	128(36) Allopathy: 88(68.7) MP: 27(21.1) Both: 13(10.2)	-	-	SM was common. Multigravidas and women with maternal illness were more likely to practice SM
Adanikin e Awoleke (2017)^(21)^ Nigeria	346 pregnant women from <20 to >40 years	Examines the burden of SM during pregnancy and the impact on fetal wellbeing	Anytime during pregnancy	79(31.5)	Paracetamol 24 (-), artesunate 10, sulphadoxine and pyrimethamine 9, chloroquine 6, clotrimazole pessary 4, amoxicillin 2 and others 27	Malaria fever 179(51.7), edema 66(19.1), headache 56(16.2), vaginal infection 49(14.2), vomiting 30(8.7) and others 146(42.4)	SM was associated with increased FDA risk category and OTC drugs need strict controls during pregnancy
Bello et al. (2011)^(22)^ Nigeria	410 pregnant women from 24 to 34 years	Assess the drug use profile, including prescribed drug compliance and SM among PW	Anytime during pregnancy	Allopathy: 78(19), MP: 190(46.3)	Hematinics 283(69), acetaminophen 196(48), anti-malarial, vitamin C and metronidazole 25(6) and calcium supplements 4(1)	-	Patients need counseling on the dangers of SM. Use of herbal concoctions needs to be explored in the community
Beyene e Beza (2018)^(23)^ Ethiopia	617 pregnant women from 18 to >34 years	Assess SM practice and associated factors among PW	Anytime during pregnancy	164(26.6) Allopathy: 112(18.2), HM: 67(10.9) and both: 15(2.4)	Paracetamol 55(49.1), amoxicillin 26(23.2), panadol 7(6.3), ibuprofen 6(5.4), albendazole 6(5.4) and others 38(34.2)	-	High prevalence of SM during pregnancy. Previous pregnancy and knowledge were significantly associated with SM practice
Jambo et al. (2018)^(24)^ Ethiopia	244 pregnant women from 18 to >35 years	Assess the prevalence of SM and contributing factors among PW	Anytime during pregnancy	170(69.7) Allopathy: 71(29.1) HM: 142(58.2)	Paracetamol - (33.8), cough syrup (23.9), do not remember (22.5), amoxicillin (18.3), metronidazole (1.4)	Allopathy: common cold 30(42.3), headache 26(36.6), nausea/vomiting 10(14.1), others 5(7.0)	High prevalence of SM. There is a need for public trainings for all women of reproductive age about the risks of inappropriate SM
Marwa et al. (2018)^(25)^ Tanzania	372 pregnant women from 18 to 27 years	Estimate the prevalence of SM and evaluated predictors of SM among PW	Anytime during pregnancy	172(46.2) Allopathy HM: 94(25.3)	Antiemetic 59(34.3), antimalarial 42(24.4), analgesics 33(19.1), antibiotics 17(9.5), cough & cold remedies 9(5.2) and others 12(6.9)	Malaria 56(32.5), morning sickness 44(25.5), headache 33(19.1), urinary tract infection 16(9.3) and others 23(15.3)	Prevalence of SM with allopathy and HM among PW was high and common among illiterate, unemployed and in the 1^st^ trimester
Yusuff et al. (2011)^(26)^ Nigeria	1594 pregnant women from 19 to 36 years	Assess the frequency and evaluate factors underlining SM with orthodox and herbal medicines among pregnant women	Last 90 days	1017(63.8) Allopathy: (58.4) HM: (31.2) Both: (10.4)	Paracetamol 485(31.1), hematinics + vitamins 365(23.4), promethazine 130(8.3), piroxicam 120(7.7), diazepam 119(7.6) and others 342(21.9)	Pain/fever 432(30.1), joint pain 208(14.5), cough 147(10.2), weakness 132(9.2), indigestion 122(8.5), headache 112(7.8) and others 283(19.7)	SM with prescription, OTC and HM is pervasive and significantly associated with gestational age and occupational pattern among studied women
Zewdie et al. (2018)^(7)^ Ethiopia	323 pregnant women from 15 to >35 years	Assess the prevalence and factors associated with SM among PW	Anytime during pregnancy	50(15.5)	Acetaminophen 29.8%, diclofenac 21% and others – (-)	Vomiting 13(25), heart burn 11(21.2), back pain 9(17.3), headache 6(11.5), constipation 6(11.5) and others 7(13.4)	Prevalence of SM was comparable to the other studies. Better maternal education and health problems were associated with SM
**CONTINENT - ASIA**							
Afshary et al. (2015)^(41)^ Iran	810 pregnant women from <25 to >30 years	Determine the prevalence and causes of SM among PW	Anytime during pregnancy	245(30.6) Allopathy: - (40), HM: (13.1), MP: (46.9)	-	Allopathy: Anti-infectious - (44.8), digestive diseases (19.3), anemia (14.2), and others (23.1)	It is necessary to take some measures raising the level of culture and preventing SM particularly in women
Atmadani et al. (2020)^(3)^ Indonesia	333 pregnant women from 16 to 45 years.	Examine the proportion of PW who self-medicated and factors associated	Anytime during pregnancy	39(11.7)	Antiemetic medicines - (33), cold and flu remedies - (29), anti-fever medication - (15), pain killers - (13), and others - (10)	-	39 women self-medicated during pregnancy. Knowledge and age were observed to be associated with the practice
Baghianimoghadam et al. (2013)^(42)^ Iran	180 pregnant women from <25 to >35 years	Determine the knowledge, attitude, and practice of PW in terms of SM	Anytime during pregnancy	63(35)	-	-	Increased prevalence of SM during pregnancy
Bohio et al. (2016)^(43)^ Pakistan	351 pregnant women from 18 to 45 years	Evaluate the frequency of OTC use among PW, type of medicines and motivation for SM practice	Anytime during pregnancy	133(37.9)	Acetaminophen 58 (43.6), acetaminophen + aspirin 24 (18), acetaminophen + ibuprofen 16 (12), multiple drugs 20 (15), ibuprofen 5 (3.8), others 10 (5.4)	Headache 80(60.2), multiple complains 26(19.5), headache + backache 14(10.5), others 13(10.1)	A significant number of pregnant women indulged in the practice of using OTC medication
Botyar et al. (2018)^(44)^ Iran	210 pregnant women and 210 nonpregnant women from 15 to 45 years	Compare SM in pregnant and nonpregnant women	Anytime during pregnancy	73(34.8) PWG Allopathy: 18(8.6) HM: 14(6.7) MP: 41(19.6)	Ibuprofen – (9), cough syrup – (8.6), and antibiotics – (8.1).	Nausea, vomiting, and heartburn - (5.7), morning sickness (4.3), lack of appetite (3.8) and others (5.3)	MP are the most common medications used by PW. They should be advised against the arbitrary use of these substances
Ebrahimi et al. (2017)^(45)^ Iran	384 pregnant women with a mean age of 26.3	Compare the prevalence of SM before and during pregnancy and its determinants factors	Anytime during pregnancy	78(20.3) During pregnancy	-	-	The prevalence of SM during pregnancy was still significant. It is necessary to provide trainings for all women of reproductive age
Liao et al. (2015)^(46)^ China	422 pregnant women from 20 to 42 years	Assess substance use and SM during pregnancy and factors associated	Anytime during pregnancy	11(2.6)	-	-	Socio-demographic parameters were associated with substance use during pregnancy
**CONTINENT - EUROPE**							
Cabut et al. (2014)^(2)^ France	60 non-pregnant women and 68 pregnant women from 18 to 45 years	Identify nonpregnant women intending to use SM during pregnancy and the proportion of PW using these products	Anytime during pregnancy	49(72.1) PWG Supplements: 15(22.7) Essential oils: 9(13.9) Herbal teas: 19(29.2)	Acetaminophen 44(89.8), phloroglucinol 14 28.6), medications for digestive disorders - 8(16.3), homeopathy 13(26.5) and others 5(10.2)	Headache 39(79.6), stomachache 18(36.7), other pains 9(18.4), nausea, vomiting 9(18.4) sore throat 8(16.3) and others 20(53.0)	Frequent intended use and actual use of SM and alternative products during pregnancy
Navaro et al. (2018)^(47)^ Italy	503 pregnant women from 15 to 44 years	Characterize knowledge, attitudes, and medications use during the pregnancy and factors associated	Anytime during pregnancy	221(43.9)	*ATC Classification:* N 180 (69.8), A 48 (18.6), M 16 (6.2), R 8 (3.1), J 5 (1.9) and C 1 (0.4)	Fever/common cold symptoms - (32.6), headache/migraine (29.5), digestive disorders (18.2), and nerve pain (15.5)	Almost half of participants practiced SM. Who were more likely to self-medicate were older, Italian, multiparous, with no history of abortion
Odalovic et al. (2012)^(32)^ Serbia	311 pregnant women from 16 to 44 years	Investigate prescription and OTC drug use among women in before and during pregnancy	6 months before pregnancy and 6 months during pregnancy	27(8.7) OTC: 23(7.4) Prescription and OTC: 4(1.3) During pregnancy	Acetaminophen - (6.4)	-	Less SM with OTC drugs was observed in pregnancy when compared to before pregnancy
Verstappen et al. (2013)^(48)^ Netherdlands	1246 pregnant women from 18 to >35 years	Describing possible predictors of OTC-medication use during pregnancy	Anytime during pregnancy	157(12.5)	Analgesics 51(27.3), vitamins 50(26.7), GI medication 40(21.4) and others 46(24.6)	-	Five predictors were included in the model. There is a need for studies that ascertain OTC use more in detail
**CONTINENT – AMERICA (North, Central and South America)**							
Alonso-Castro et al. (2018)^(28)^ Mexico	1798 pregnant and non- pregnant women that got pregnant in the last 3 years, from <25 to >35 years	Evaluate the prevalence and the factors associated with SM among women	Anytime during pregnancy	393(21.9) Allopathy: 110, MP: 264, other products: 76	Paracetamol 83(42.8), bonadoxin 16(8.2), another NSAID 12(6.2), omeprazole 10(5.2) and others 139(34.4).	Migraine 57(51.8), nausea 28(25.5), gastritis 22(20), cold 18(16.4), constipation 15(13.6) and others 41(37.2)	SM is common among PW. Adequate counselling of PW about the potential risks of SM drugs during pregnancy is strongly warranted
Araújo et al. (2013)^(27)^ Brazil	78 puerperal women, from 10 to >20 years	Evaluate the use of medicines during pregnancy and the factors associated	Anytime during pregnancy	22(28.2)	Analgesics 16(20.5), anti-inflammatory and antirheumatic 3(3.9), vitamins 2(2.6) and others 8(10.4)	-	The practice of SM exists, even with access to health. SM was related to PW with lower number of antenatal visits and smokers
Bercaw et al. (2010)^(49)^ USA	485 puerperal women from 18 to 42 years	Assess drug use among PW (herbs, vitamins, OTC and prescription medications)	Anytime during pregnancy	OTC: 112(23) HM/ vitamins: 313(64)	Acetaminophen 63(13), Robitussin 28(6), Maalox 22(5), Ibuprofen 17(4) and others 38(9)	-	Use of HM does not appear to be a replacement for conventional medicine among most PW. Patient education is necessary
Miní et al. (2012)^(50)^ Peru	400 pregnant women from 19 to >40 years	Evaluate the prevalence and possible causes of SM among PW	Anytime during pregnancy	42(10.5)	Acetaminophen - (47.6), amoxicilin (16.7), ibuprofen (16.7), naproxen (11.9), dimenitrate (4.8) and vitamines (2.4)	Pain - (40.5), respiratory problems (28.6), urinary tract infection (16.7), fever (9.5) and nauseas (4,8)	Low prevalence of self-prescribing behavior during pregnancy compared to the international literature
Rocha et al. (2013)^(6)^ Brazil	326 puerperal women between 13 and 45 years	Evaluate the use of medications, alcohol and smoking during pregnancy	Anytime during pregnancy	37(11.3)	Anti-inflammatory 21(39.6), Analgesic/ antipyretic 13(24.5) and others 19(35.8)	-	Being single was found to be a risk factor for exposure to high teratogenic potential

SM - Self-medication; PW - Pregnant women; Missing information; MP - Medicinal Plants; HM - Herbal Medicines; OTC - Over-the-Counter; GI - Gastrointestinal; ATC - Anatomic Therapeutic Chemical Classification System. All studies have a cross-sectional design, excepted Beyene e Beza^(23)^ and Odalovic et al.^(32)^

Patients and public were not involved in this study.

## Results

**Figure 1:**
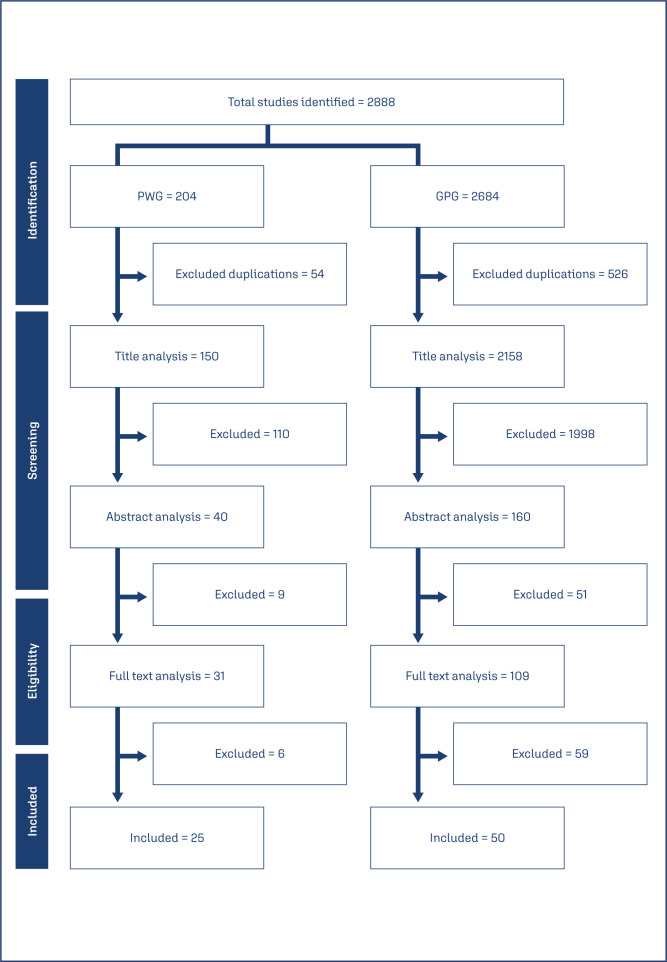
Flow chart for the screening and selection process. PWG: Pregnant Women Group; GPG: General Population Group.

The literature search identified 2888 articles, classified as 204 and 2684 articles for PWG and GPG, respectively. Duplications were excluded using EndNote® Software and Rayyan's web application.^(40)^ The studies were evaluated by two independent reviewers and the discrepancies were resolved by a third independent reviewer. Studies were excluded due different reasons, including wrong population and outcome, use of specific drug and wrong study design. Finally, 75 studies were included after full review (25 from PWG and 50 from GPG). The screening and selection process is described in figure 1.

### Country of publication

For the *Pregnant Women Group (PWG),* the publications were from 15 countries distributed in all 6 continents. The publications were higher in low-income countries (44%), followed by middle (40%) and high-income countries (16%). In the *General Population Group (GPG),* the publications were from 28 countries distributed in all 6 continents. Middle-income countries lead the number of publications (40%), followed by low (36%) and high-income countries (24%).

### Sample size and population

In the PWG, the sample size ranged from 78^(27)^ to 1798^(28)^ participants, with 13100 women enrolled. The most studied population was current pregnant women, followed by puerperal and reference non-pregnant women (compared to pregnant women or women that got pregnant in the last 3 years). According to women's age, 12 studies considered women older than 18 years, 8 studies included women younger than 18 years and 5 studies had no age restriction in the inclusion criteria. For the GPG, the sample size ranged from 138^(29)^ to 31573^(30)^ participants, with 95459 people enrolled. According to participants’ age, all studies considered participants older than 18 years and 9 studies focused on the elderly population. In relation to sex/gender, one study was performed considering only the male population^(31)^ and the remaining studies considered male and female population.

### Study design

In the PWG, twenty-three studies have a cross-sectional design, while one study used a cohort design^(32)^ and another was a mixed methods study (cross-sectional with qualitative study).^(23)^ For the GPG, forty-eight studies have a cross-sectional design, one is a mixed method study (a cross-sectional with qualitative section study)^(33)^ and one is a cohort study.^(34)^

### Recording period

There was a small difference regarding the recording period considered to assess SM practice in the PWG. Almost all studies considered self-medication during the whole pregnancy period, while one study considered SM in the first six months of pregnancy^(32)^ and one study used the past 90 days before the survey.^(26)^ In the GPG, the period considered to evaluate practice varied between days and years. The shortest period considered was the last three days prior to survey^(31,35)^ and the longest was the last 12 months.^(10,33,36–38)^ The recording period considered to assess SM practice was not clear in eight studies. Some studies considered more than one period^(39,51)^ or used an unspecified time period.^(52)^ Eight studies considered medications used at the study moment as recording period.

### Definition of self-medication

In both groups, there were no consensus regarding the definition of self-medication. Some studies considered SM only as the use of over-the-counter drugs (OTC), while other studies considered OTC, prescribed drugs, herbal medicines, medicinal plants and other types of Complementary Alternative Medicine (CAM). The definition of SM was not described in some studies. The definitions of SM during pregnancy considered in each study are displayed in chart 1.

### Self-medication prevalence

In the PWG, SM prevalence ranged from 2.6^(46)^ to 72.4%^(19)^ and most studies had the SM prevalence between 21% and 50%.^(20,21,23,25,27,28,41–44,47)^ For the GPG, the SM prevalence ranged between 8.9^(35)^ and 100.0%^(53–55)^ and most studies had the SM prevalence higher than 50%.^(10,36–39,52,53–75)^ The general SM prevalence was not reported in one study^(33)^ and in another study was reported by the frequency of medications purchased.^(29)^ The cohort study reported SM's prevalence throughout the studied years^(34)^ and one study evaluated SM's practice in two time periods (during life and in the last 30 days).^(51)^ Two studies evaluated two sub-populations (urban and rural residence)^(76)^ or more than one aim (SM for general and for rheumatic symptoms).^(77)^

### Exposure period to self-medication during pregnancy

The exposure period to SM during pregnancy was reported only in 8 studies. Seven reported the SM practice according to each trimester and one study reported only the trimester with higher prevalence of SM. The studies and prevalence data on the exposure period to SM during pregnancy are described in chart 2. None of the 8 studies stratified the medications according to gestational trimesters.

**Chart 2:** Exposure period to self-medication during pregnancy according to gestational trimesters

Author	Trimester		
	1^st^ n(%)	2^nd^ n(%)	3^rd^ n(%)
Afshary et al.^(41)^	41(41.8)	49(50.0)	8(8.2)
Atmadani et al.^(3)^	4(10.3)	35(89.7)*	35(89.7)*
Beyene e Beza^(23)^	122(-)┼	96(-)^┼^	17(-)┼
Marwa et al.^(25)^	59(34.3)	87(50.5)	26(15.1)
Navaro et al.^(47)^	45(17.4)	121(46.9)	43(16.7)
Odalovic et al.^(32)^	1(0.3) / 13(4.2)^i^	3(1.0) / 17(5.5)	Missing
Rocha et al.^(6)^	Higher in first trimester	-	-
Zewdie et al.^(7)^	15(30.0)	- (40)	- (30)

* Value 35 (89.7) related to SM in the 2nd and 3rd trimester; - missing information;

┼ total obtained from SM with allopathy and MP; Ø authors considered SM until 2nd trimester;

i SM with prescribed and OTC/ SM just with OTC

### Medications used during self-medication

In the PWG, medications were classified according to the medication name (n = 7), medication group (n = 6), drug name and/or medication group (n = 3), medication name and Food and Drug Administration Risk (FDA) (n = 2), drug name and Anatomic Therapeutic Chemical Classification System (ATC) (n = 1), just ATC (n = 1) and 5 studies did not report the medications used in SM practice. Acetaminophen was the most used medication and studies that considered the medication group, analgesics were the most used medications followed by antiemetics. For the GPG, twenty-five studies reported the drugs used according to the medication group. Sixteen studies did not report the medications used during SM practice and other studies reported according to drug name and ATC classification. The most used medication group were analgesics (n = 12) and antibiotics (n = 4). Cough and cold preparations were predominant in just one study. Acetaminophen was the most used medication, followed by metamizole. Studies that considered the ATC classification reported as the most used medications - musculoskeletal and nervous system.

### Symptoms and self-medication

The symptoms triggering SM initiation in the PWG were missing in thirteen studies. Headache (n = 3), pain and fever (n = 3), malaria fever (n = 2), nausea and vomiting (n = 2), common cold (n = 1) and infections (n = 1) were the most common symptoms that prompted SM during pregnancy. For the GPG, the symptoms that triggered SM practice were missing in twenty-nine studies. Headache (n = 10), fever (n = 3), flu, cold and cough (n = 4), gastrointestinal problems (n = 2), pain (n = 1), allergies (n = 1) and musculoskeletal problems (n = 1) were reported as the main causes of SM practice.

## Discussion

In this review, we summarized and compared self-medication patterns in pregnant women compared to the general population. The reported average of self-medication prevalence among pregnant women was between 21%-50%, considerably lower than the reported average among the general population; with prevalence rates surpassing 50% in most of the studied populations. Seventy-five studies were included in this review (majority from GPG) with a total of 108,559 participants. Most of the studies had a cross-sectional design – as expected - and were most from low-income and middle-income countries. The recording period considered, and the definition of SM was different between the studies. Acetaminophen was the most used medication in both groups and headache was the leading symptom related to SM practice.

The PWG was the group with the smaller number of studies and total participants interviewed in this review. It can be justified by the fact that studies with pregnant women have limited inclusion in comparison to general population. From all studies included, two of them used a mixed-method approach.^(23,33)^ According to Van der Geest,^(78)^ this design can provide a complete understanding of the phenomenon (in this case, self-medication) as the qualitative segment could bring crucial patient perspectives that were missed through the use of quantitative interviews only.

The SM prevalence changes according to the recording period considered.^(79)^ Almost all studies in the PWG used the entire pregnancy as recording period, while in the GPG it varied from 3 days^(31,35)^ to 1 year.^(10,36–38)^ Although the literature shows that longer periods can contribute to recall bias,^(78,80)^ studies in the GPG that considered long periods (over 2 months) had a higher SM prevalence (37.8 to 94.9%) compared to studies with shorter periods (8.9 to 57.0%).

Reports suggest that the difficulty in memorizing increases with age^(79)^ and SM is practice for minor illness can be rapidly forgotten.^(78)^ Studies with elderly population included in this review considered shorter time periods^(34,35,60,66,67)^ due to population's profile.^(80)^ Considering the most common symptoms that leaded to SM in PWG, there also can be a lack of notification among this group. According to the literature, there is no ideal period considered to assess the SM practice but two-week period is recommended and it will depends on the study goal.^(80)^

The SM prevalence was higher in the GPG and it can be attributed to the population's profile, recording period assessed and the considered SM definition. The three studies in the GPG that had a SM prevalence of 100% included only people who self-medicated^(55)^ or people that were buying the medicines to self-medicate.^(53,54)^

In the PWG, the higher SM prevalence was reported in the second trimester in six of the eight studies. Although the first trimester is more critical regarding potential damage during fetus development, this risk can reach the fetus throughout pregnancy by different ways according to the exposure period.^(81)^ Beyond the fetal risk, women are exposed to the risks of SM practice, including Drug Related Problems (DRP), delay in diagnosis, allergies and intoxication.^(15,82)^

Acetaminophen was the most used medication in SM by pregnant women. Although it is the first choice as analgesic during pregnancy, some studies suggested an association between prenatal exposure to acetaminophen and an increased risk of behavioral problems in childhood.^(83–86)^ The use of non-steroidal anti-inflammatory drugs (NSAIDs) was also common. NSAIDs (e.g. naproxen) are known to cross the placenta^(87)^ and studies show that when administered in early pregnancy, they can be associated with oral cleft, neural tube defects, encephalocele and spontaneous abortion.^(88–90)^

Beyond the risk of teratogenic effects, SM during pregnancy can delay an important diagnose, that may lead to premature birth and other serious consequences to the infant. The finds of this review reinforce the need for expanded control the use of medications during pregnancy, even if it is proven to be safe regarding malformation aspect and shows the importance to promote a non-pharmacological treatment during pregnancy when a diagnose is established. Cognitive behavioral therapy, acupuncture, yoga and massage showed promising effectiveness in treating mild problems like perinatal anxiety, pain and insomnia during pregnancy,^(91–93)^ warranting additional larger studies.

The absence of the medications used during SM was less common in the PWG (12.0%) when compared to the GPG (32.0%). Three studies^(47,60,94)^ classified the medications used according to the ATC classification system. According to this classification, we recommend classifying using a fourth level, as classification using three levels,^(47,94)^ is inadequate to assess which medication was used as the same class can have medications with different risk levels during pregnancy.

Regarding the symptoms that prompted the SM practice, the absence of this information was similar in both groups, missing in 52.0% and 58.0% in the PWG and GPG, respectively. Since SM is generally practice treating minor illness, it can be difficult to assess the symptoms that motivated its practice, once they can be rapidly forgotten. In the studies that this information was considered, the symptoms reported were similar between groups and the primary indication was headache.^(78)^

### Strengths and limitations

- The search used included articles published in the last 10 years and studies performed before this period were not considered.
- Although WHO proposes a universal definition for SM, this definition change between the studies, preventing comparison of studies within the literature.
- This review provides a comprehensive overview of SM during the last 10 years in different populations.
- The results reported can help to improve health polices and promote rational medication use.

## Conclusion

This scoping review identified that self-medication is common among pregnant women, however relatively less frequent when compared to the general population. The medications used in self-medication and their indications were similar between the groups. There was a significant difference in the definitions used to describe self-medication, highlighting the need for a universal update for this concept.
